# Ten tips for conducting focused ethnography in medical education research

**DOI:** 10.1080/10872981.2019.1624133

**Published:** 2019-05-31

**Authors:** Marghalara Rashid, Carol S. Hodgson, Thea Luig

**Affiliations:** a Department of Pediatrics, Faculty of Medicine and Dentistry, University of Alberta, Edmonton, Alberta, Canada; b Department of Family Medicine, Faculty of Medicine and Dentistry, University of Alberta, Edmonton, Alberta, Canada

**Keywords:** Medical education, ethnography, focused ethnography, methodology, qualitative methods

## Abstract

**Background**: Medical education researchers increasingly use qualitative methods, such as ethnography to understand shared practices and beliefs in groups. Focused ethnography (FE) is gaining popularity as a method that examines sub-cultures and familiar settings in a short time. However, the literature on how FE is conducted in medical education is limited.

**Aim**: This paper provides 10 practical tips for conducting FE in medical education research.

**Methods**: The tips were developed based on our expertise in ethnographic research and existing literature.

**Results**: The 10 tips include: (1) Know the difference, (2) Build relationships before you start, (3) Have shared purpose and knowledge translation strategies with your stakeholders (4) Practice being reflexive, (5) Align research question with methodology, (6) Prepare your fieldwork, (7) Use a variety of methods for data collection, (8) Consider context on micro, meso, and macro levels, (9) Use triangulation, and (10) Provide a ‘thick description’,

**Conclusions**: These 10 tips give practical guidance to medical educators in thinking about how and when to conduct FE.

## Introduction

For many health professions education (HPE) researchers, there is an increasing desire for a richer and deeper understanding to the questions they find in their everyday clinical practice. The use of qualitative methods is trending in medical education, which was pointed out in recent medical education publications [1,2]. Qualitative methodologies have advanced our knowledge by providing insightful accounts of the socio-cultural factors that impact the development, delivery, and outcomes of medical education [3]. Ethnography, for example, has a history of more than 50 years in medical education [4]. Examples of ethnographic studies that impacted medical education include *The Student Physician* by Fox from 1957 [5], which explored uncertainty in medical knowledge, and the 1961 landmark ethnographic study entitled *Boys in White* that examined the culture of medical students, their every day lives, and interactions in medical school [6]. Ethnography as a research method has elicited a valuable body of literature in this area [4] by observing, inquiring, and understanding peoples’ experiences, interpretations, their interactions, and relationships surrounding a topic in a real-life context. In this paper our focus is to introduce a recent form of ethnography, commonly referred to as focused ethnography (FE), that is gaining popularity in medical education [7], health research [8,9], and nursing education [10].

Ethnography is the description of people and their way of life. As a key method of anthropology, ethnography is concerned with culture, shared practices and beliefs, and how the social context shapes, and is shaped by, individuals [11]. Historically, ethnographers have studied unfamiliar cultures and spent extended periods in the field. Ethnographers immersed themselves in culture by learning the language and taking part in day-to-day activities. Today, researchers still value ‘cultural immersion’ to achieve a deeper understanding of the insider’s perspective through long-term fieldwork [12]; however, the field can be anywhere in the world including contemporary societies and familiar settings. Field data are collected using multiple methods such as participant observation, interviewing, document reviews, visual methods, or studying cultural artefacts. Ethnography differs from other qualitative methods because of its use of participant observation to understand shared meanings and practices. People do not or cannot always describe what they actually do and think during an interview. Observation and participation in context, however, can provide insights about real-world social and cultural processes that shape outcomes of interventions in medical education. As such ethnography can be invaluable to answer emerging questions in health-care settings; however, it needs to adapt to the specific context of medical education research [10].

Over the years, ethnography as a methodology has changed and, in response to the specific needs and contexts of different fields of research, contemporary forms of ethnographic practices, such as focused ethnography, have been developed [13]. Focused ethnography (FE) emerged within research contexts where long-term fieldwork is less often feasible. There is consensus among researchers today that FE is a well-suited methodology to examine sub-cultures within modern complex societies [14,15], such as interprofessional ward teams, teams in the emergency room during a resuscitation, or nurses in the neonatal intensive care unit (NICU). As ‘the study of shared experiences of a more confined, predetermined phenomenon’ [16] p.3 FE collects context-sensitive and culturally appropriate data in an efficient, pragmatic, and rapid way.

While FE offers a more pragmatic and feasible method for the medical education research context, the field of medical education has largely had a positivist approach to research and training that is only recently moving towards more interpretivist approaches (naturalistic inquiry). Researchers have come to acknowledge the importance of investigating the ‘how’ and ‘why’ research questions [17] including aspects of workplace or education culture. As a result, there is a gap between research practise that increasingly uses qualitative approaches, including FE, and the availability of training and literature on how to conduct high-quality focused ethnographies. In this paper, we provide 10 practical tips to help educators and researchers in medical education to design a FE project. Furthermore, we have organised these tips in the order of how one might think about different aspects of a FE project. In reality, the aspects addressed in the tips will be important at various times during the project, and will need consideration iteratively or simultaneously with others.

## Tip 1

### Know the difference

Understanding the difference between traditional ethnography and focused ethnography (FE) will allow researchers to choose the appropriate form of ethnography in order to explore their research question rigorously. Traditional and focused ethnography share many common features and require similar techniques to ensure quality data and analysis. The main difference lies in pragmatic considerations and what is feasible for the context of a medical education research project. In traditional ethnography, there is an emphasis on continuous and long-term fieldwork. The scope is generally vast and requires a commitment to gain an insider’s perspective. Ethnographers aim to familiarize themselves with the culture by actively participating, learning the language, and living alongside local people.

In contrast to traditional ethnography, FE explores a specific problem within sub-cultures and among small groups of people [18]. In recent years, scholars have found FE to be valuable and effective for technologically intricate modern sub-cultures [19]. Because, the scope of FE is narrow, the researcher generally has greater knowledge about the topic under study and relies less on immersion in cultural practices and interpretations by engaging in long-term fieldwork. Thus, FE is more feasible for busy medical educators who are curious and notice interesting interactions or outcomes in their own setting or other relevant groups that they may be interested in exploring. Focused Ethnography applies to any small-scale research that is conducted in the everyday setting, explores shared practices and meanings from a cultural lens, and where the researcher may or may not have familiarity with the sub-culture under study. FE is problem focused and context specific [20].

## Tip 2

### Build relationships before you start

Focused ethnography is about understanding people. Understanding people requires relationships; building relationships takes time. Starting early, before the official beginning of data collection, and getting to know a community or population group in context is helpful in a number of ways. Implementing a FE project benefits from partnering with the people affected through all stages. Informal interactions and building relationships with leaders or representatives of a professional community or population group, can help researchers acquire some familiarity with the context of the population, which allows for ‘exploration, reflexivity, creativity, mutual exchange and interaction’ [21] before research design and ethics applications are completed. The purpose of this engagement is to refine the project and methods. It is not data collection, in fact, nothing learned during these informal encounters is data without an appropriate ethics approval from a relevant academic institution. Investing this time can provide invaluable information to design a relevant and useful project. Informal conversations can help understand whether the research builds on an appropriate grasp of the issue(s), whether research questions are relevant, and help identify key informants and potential interviewees [22]. Listening and observing can help find out what is polite to ask and what is not. It can give a sense of how acceptable certain methods may be. Relationship building before completing the study design is a form of stakeholder engagement, and recognizes that key stakeholders of medical education research include learners, practitioners, and patients.

## Tip 3

### Have shared purpose and knowledge translation strategies with your stakeholders

The definition of knowledge translation (KT) by the Canadian Institutes of Health Research (CIHR) [23] highlights the exchange and application of knowledge to improve the health of people. Important for this is bi-directional knowledge transfer is collaborative decision-making between the research team and stakeholders on a number of issues: (1) which questions to answer; (2) how to answer them; and (3) how to disseminate and implement recommended findings. Such partnerships provide a platform to share and discuss project progress, key findings, and to ensure recommendations are relevant to policy and systems issues. The goal of KT goes beyond academic publishing or presenting research; it emphasizes dissemination of the research results directly to the stakeholders and community partners. When developing a KT and dissemination plan before the start of a project, research team members and stakeholders anticipate together clear, practical steps to be used during and after project completion that can lead to improved services and policies. With an eye on implementation, the researchers should ask a number of questions. Why am I doing this? What is the ultimate goal of conducting this research project? Is this project transferable to the real world? For whom is the knowledge intended? How should the emerging knowledge be transferred? [24]. Exploring these questions in partnership with stakeholders fosters research that has the potential to change practice and polices with positive outcomes for teaching and care delivery.

## Tip 4

### Practice being reflexive

During the 1980s, ethnographic practice underwent a tremendous shift recognizing how the cultural, socio-economic, and historical *position* of researchers and their disciplines shapes encounters in the field, as well as the interpretation of data and representation of knowledge in ethnographic writing [25]. As a result, *reflexivity* became a key practice in ethnography that helps researchers become aware of, make transparent, and transcend personal perspectives and biases that impact research relationships, researcher perceptions on how data are collected and interpreted. Reflexivity is introspection and reflection about how and why we as researchers and participants think the way we think, what we pay attention to, what we overlook and take for granted, how we ask questions, interpret answers, and represent results in writing. It is about bringing awareness to how our perception, thinking, and perspective is dynamically shaped by our culture, age, gender, social status, personal history, language, values, and experiences [26]. This applies to both, FE researchers who are familiar with the group under study and researchers who enter a field unfamiliar to them. Reflexivity is essential and acknowledges that no researcher can take a totally objective stance in understanding and describing another or one’s own culture or human group.

**Figure 1.**
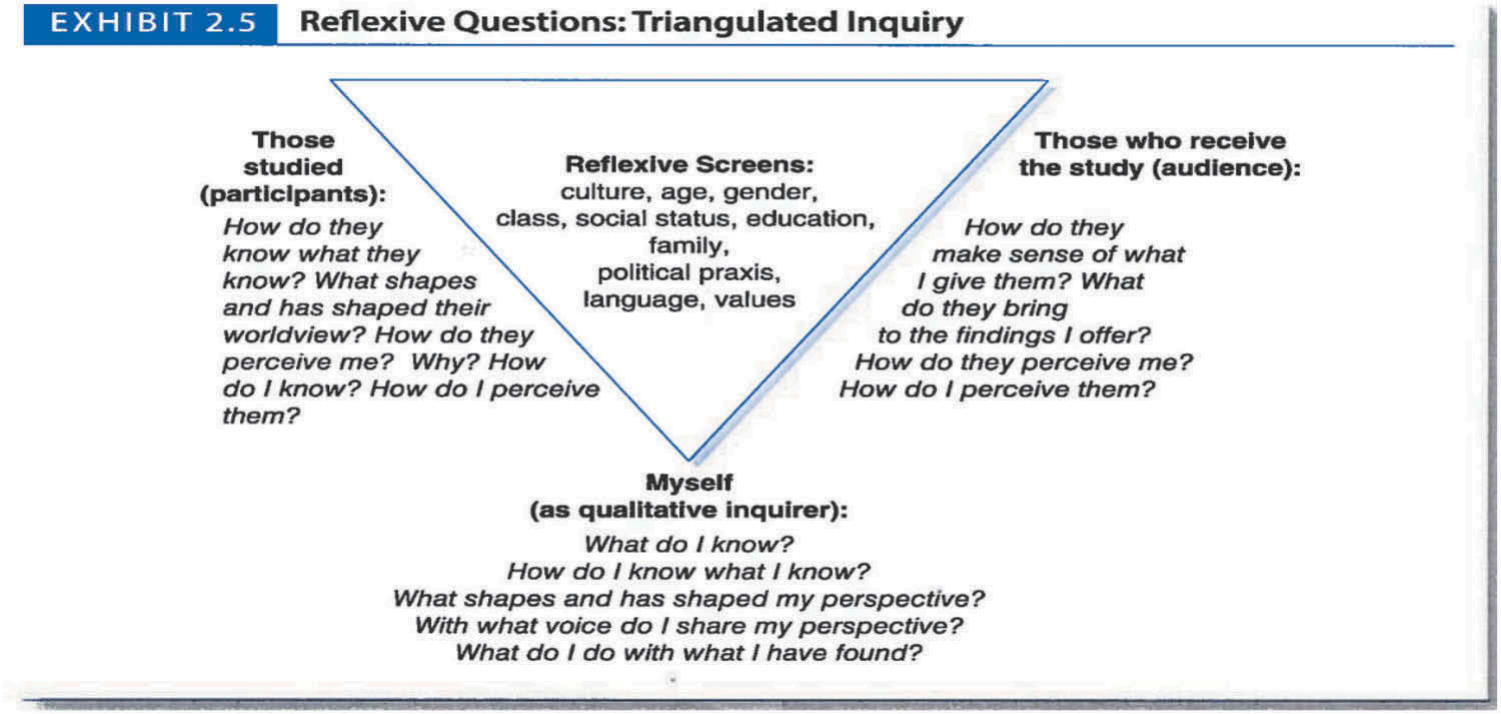
Reflexive Questions: Triangulated Inquiry.

To practice reflexivity, Patton suggests a triangulation of reflexive questions (Patton 2015, see Figure 1) [11]. As with every skill, reflexivity needs practicing. Ask yourself: Why and how did you become interested in your topic? How did you adopt your theoretical framework? What values and feelings do you associate with the topic and people you are working with? What do you want to achieve? Why do you think it has value and for whom? What do you take for granted, What do you pay attention to? What could you overlook because of your existing perception of the problem? The goal is to reflect critically on one’s own role and the role of others in shaping the knowledge created with research.

Patton M, Qualitative Research and Evaluation Methods (4th edition) pp. 72. Copyright © [2015] (Sage Publications, Inc.). DOI: [DOI number 978–1-4129–7212-3].

## Tip 5

### Align research question with methodology

Just as in quantitative research and other qualitative research, FE research starts with a solid research question, which is the foundational building block of an entire research project. Your research question will guide the study design and methodological choices of your research team [27]. It is vital that a strong cohesion exists between the research question, methodology, data collection, and theoretical framework. Lack of alignment deteriorates the overall credibility and authenticity of a research study. Choosing a theoretical lens that explains constructs and relationships relevant to answering the research question is vital and helps novice researchers have a clear understanding of what to focus on during data collection and analysis. Making ones’ theoretical lens explicit helps others critically evaluate the research and provides a rational for the choices of research methods and strategies for data analysis. Further, a thorough review of the existing literature will determine if the research question is novel. In FE, research questions are about understanding experiences of individuals, shared practices and meanings within a sub-culture or specific population. For example, what are the shared values and beliefs of faculty members, medical students, and community members who sit on medical school admissions committees? How do cultural elements of the group affect individual and collective behaviours, decisions, and outcomes? As a foundation for and guide throughout the research process, the research question should be precise and specific, clearly outlining what is being investigated and who is the study population. In FE like in quantitative research, research questions should be FINER [28], that is feasible, interesting, novel, ethical, and relevant.

## Tip 6

### Prepare your fieldwork

Many ethnographers have stories to tell about once-in-a-lifetime events when their recording device failed. Accidents happen even with the best preparation; however, thoughtful organization of technologies, supplies, and logistics can help data collection succeed [29]. Below are some pragmatic tips usually not included in the published literature on methods.

#### Technology

Reflect on how much equipment is manageable to carry during your observations and activities. Invest in a quality digital audio or video recorder. Learn how to use your equipment beforehand. Test equipment *before* you go in the field. Bring extra batteries and memory cards; remember all cables and adapters. Make a checklist to help gather all pieces before leaving for an interview or field visit. Become knowledgeable about capturing sound and microphones for different environments and purposes. Find out if interviews can take place in a quiet space or if you need to capture voices in noisy surroundings where you may need different recoding devices. Practice recording until you achieve useable recordings.

#### Interviewing

Plan enough time before and after interviews to reflect, catch your breath, and gather your thoughts. Keep a binder that contains all documentation, such as consent forms, information letters, interview guides, and receipt booklets for honoraria. Reflect on how participants relate to different spaces. How does the space affect how people feel, speak, and, as a result, shape the data collected? Plan for unusual interviewing places. At the beginning of each interview, state the date, location, and participant ID on the recording. Double-check that your devices are indeed recording. Interviewees may take the conversation in unexpected, yet valuable directions, be prepared to interview about these on the fly. It is useful to practice with colleagues and friends. Try not to interrupt too soon and too much. Stay with silences, avoid pushing the conversation by taking over and talking. Silences are important.

#### Note taking

Think about if one or two notebooks for field notes, interview notes, and personal reflections suits you best. If you prefer a laptop or tablet consider whether the setting and activity will allow you to plug-in or re-charge a device, whether using technology will hinder you in following along with participants, and how it may affect conversations and relationships. If recording an interview, it may be better to stay present in the conversation, maintaining eye contact (if appropriate), rather than taking notes. After the interview is over, write down your observations, non-verbal cues, emotional dynamics, your immediate thoughts and interpretations. Back up your recordings as soon as you can. Back up. Back up!

#### Time

Plan extra time: even if the question, context, and group of people is specified and bounded in FE, research with people in context often takes unexpected turns and requires more time.

## Tip 7

### Use a variety of methods for data collection

FE is conducted over a short period and fieldwork is often conducted in intervals. The researcher will only be in the field for specific times to observe certain events [13] whereas in traditional ethnography fieldwork is ongoing and conducted over a long period. Building on your early engagement in the field (Tip 2), field visits will allow researchers to develop trustable relationships with their participants, which is crucial to gain a deeper understanding of interactions and meanings with the particular sub-culture. In FE, it is important to use multiple data collection methods. Data collection is intense and includes interviews, short field visits conducted occasionally, observations, field notes, audiovisual recordings, document reviews, and archival research [13].The rational for using multiple data collection is to maintain rigour and to gather in-depth data about the topic under study.

Field notes consist of facts, such as the participants in attendance, time and date of the observation, detailed description of activities observed, who is present, and what is happening. Field notes also consists of researchers’ thoughts and feelings, interpretations, and reflections on biases.

During an interview, it is important to provide and promote an atmosphere where participants share their thoughts and feelings freely [30]. Using a variety of probes is encouraged to elicit more detailed answers; a nuanced understanding; and to capture higher quality data. The key is to ask open-ended and non-judgmental questions. The interviews will involve an in-depth exploration of how meanings are derived through interactions in a particular sub-culture. Usually, the interviews last 45–60 min and the data are collected at the location preferred by the participants.

Observations in FE are conducted in short intervals at a selected time frame as opposed to long term and continuous. Observation is significant for studying the day-to-day activities of study participants more closely. In FE field observation, the researcher does not actively participate, but merely observes participants, interactions, or events [31]. Field observation will give rich contextual data and enhance our understanding by getting first-hand information about the culture or sub-culture being studied.

In FE, as in any qualitative methodology, data collection and data analysis happen simultaneously. Recordings are transcribed verbatim, and data may be entered in software, such as N-Vivo, to facilitate data analysis. Researchers should immerse themselves into their data and be actively involved in taking analytical notes throughout their data analysis.

## Tip 8

### Consider context on micro, meso, and macro levels

Ethnography in healthcare is often used to understand the *context* of a specific problem, but what is *context*? Where does it start and end? The three levels of analysis, micro (e.g. learners, patients, educators), meso (e.g. organizations, groups), and macro (e.g. historical, political-economic, societal), are a useful structure, for both quantitative and qualitative research [32]. To consider how the issue under investigation is shaped by and shapes individuals, collectives, organizations, and socio-economic structures [33]. Relationships are at the centre of the transmission of knowledge and practices. Individuals (micro) relate to others within a group or a team but also outside of it. Groups as a whole and members of a group individually are connected to other groups in multiple, overlapping ways (meso). These interactions happen within social and socio-economic structures (macro). Within this context, some individuals and groups have more power to shape what is normal, possible, valued, and known. The levels of analysis are nested within each other as each relates to the other. While data collection and analysis in FE may focus on the micro level, it is crucial to contextualize and interpret findings in light of their intersections with meso (e.g. how admissions processes favour or disadvantage applicants) and macro processes (e.g. how funding and policies contribute to health disparities). These intersections will begin to emerge during data collection and ongoing analysis; and the insights may shift data collection and analysis iteratively to further explore how processes and outcomes shape each other at all three levels. It is especially important, when FE aims to inform change in medical education, to consider interactions between the individual, collective, and structural, systemic levels and how they affect knowledge, values, and practice and their change.

## Tip 9

### Use triangulation

The most important method to establish trustworthiness and credibility in FE is triangulation. Triangulation allows researchers to examine the data from different viewpoints and to check their interpretations against potentially disconfirming information. There are different types of triangulation:

(1) *Method triangulation* is the combined use of different methods, in FE it will include interviews, participant observation, field notes collected during short field visits, and documents [34]. This helps researchers to compare and contrast across different sources of data to confirm findings [20].

(2) *Data source triangulation* involves collecting data from different participants in different settings and at different time points [30]. Further, it is recommended that the research team take their analysed data back to the participants to make sure that the data reflects their experiences [35]. Having a diverse set of data will increase the overall quality and rigour of the study outcomes.

(3) *Investigator triangulation* involves different researchers to crosscheck data, i.e. validating transcripts, and analysis, i.e. cross coding, and critically question interpretations. Investigator triangulation adds greater depth and breadth to the credibility and trustworthiness of the data [36].

(4) *Theory triangulation* means utilizing a number of relevant theories for exploring and interpreting the data. This helps in supporting, explaining, or questioning study findings.

## Tip 10

### Provide a ‘thick description’

One of the main principles of credibility in ethnography, including FE, is to provide *a thick description. Thick description* refers to a comprehensive description of the setting, events, relationships, physical environment, people, and phenomena encountered in field work. Geertz (1973) [33] who coined the word ‘thick description’ believed that comprehensive description of ethnographic research led to the thick interpretation of inquiry, which helps understand intricacies of cultures and possible alternative meanings [37]. It includes an interpretation of the meaning attached to what was observed through an understanding of culture, history, social relations, and participants’ feelings and emotions [38]. Thick description is a backbone for constructing knowledge and interpreting complex cultural symbols and human interactions. Thick description including participants’ voice and stories in verbatim quotes, mediated by the researchers’ interpretation and theoretical lens will allow other researchers to draw conclusions about the phenomenon being studied and enhance the overall credibility and authenticity of a research project [35].

## Conclusion

We presented practical tips to prepare the reader to conduct FE in health profession education. Focused ethnography emphasizes fast and intense data collection, sometimes this means that the importance of fieldwork is neglected. Capturing complexities within a sub-culture is the core of FE. For that, being present in the field and building relationships, even for shorter periods and for selected events, is crucial. For a successful research project, field data cannot be compromised at the expense of swift data collection, which will impair the overall value of a research project. Although FE explores familiar sub-cultures and has a smaller scope, it should maintain the major feature of its parent methodology – ethnography – that is fieldwork. This will allow for a thicker, deeper and more accurate understanding of the complex and multilayered nature of relationships within a specific sub-culture and its context.

## References

[1] Ramani S, Mann K. Introducing medical educators to qualitative study design: twelve tips from inception to completion. Med Teach. 2015;21:1–7. 10.3109/0142159X.2015.1035244 25897710

[2] McGrath C, Palmgren PJ, Liljedahl M ‘Twelve tips for conducting qualitative research interviews’. Med Teach. 2018;1–5. 10.1080/0142159X.2018.1497149 30261797

[3] Reeves S, Peller J, Goldman J, et al Ethnography in qualitative educational research: AMEE guide No. 80. Med Teach. 2013;35(8):e1365–e1379. 23808715 10.3109/0142159X.2013.804977

[4] Goodson L, Vassar M An overview of ethnography in healthcare and medical education research. J Educ Eval Health Prof. 2011;8:1–5. 21637319 10.3352/jeehp.2011.8.4 PMC3100516

[5] Fox R Training for uncertainty In: Merton RK, Kendell PL, editors. The student physician. Cambridge (MA): Harvard University Press; 1957. p. 207–241.

[6] Becker HS, Geer B, Hughes EC, et al Boys in white: student culture in medical school. Chicago (IL): University of Chicago Press; 1961.

[7] Steinert Y, Basi M, Nugus P How physicians teach in the clinical setting: the embedded roles of teaching and clinical care. Med Teach. 2017;39(12):1238–1244. 28830280 10.1080/0142159X.2017.1360473

[8] O’Mahony JM, Donnelly TT Using critical ethnography to explore issues among immigrant and refugee women seeking help for postpartum depression. Issues Ment Health Nurs. 2012;33:735–742. 23146007 10.3109/01612840.2012.701707

[9] Gagnon AJ, Carnevale F, Mehta P, et al Developing population interventions with migrant women 14 international journal of qualitative methods for maternal-child health: a focused ethnography. BMC Public Health. 2013;13:471–485. 23672838 10.1186/1471-2458-13-471 PMC3733625

[10] Wall S Focused ethnography: a methodological adaptation for social research in emerging contexts. Forum Qual Soc Res. 2015;16:1.

[11] Patton M Qualitative research and evaluation methods 4th edition. Thousand Oaks (CA): Sage Publication; 2015.

[12] Richards L, Morse JM Readme first for a user’s guide to qualitative methods 2nd edition. Thousand Oaks (CA): Sage Publications; 2007.

[13] Knoblauch H Focused ethnography. Forum Qual Soc Res. 2005;6:44–58.

[14] Atkinson P, Hammersley M Ethnography and participant observation In: Denzin NK, Lincoln YS, editors. Strategies of qualitative inquiry. London, UK: Sage; 1998 p. 110–136.

[15] Fetterman MD Ethnography: step-by-step. Thousand Oaks (CA): Sage; 2010.

[16] Rashid M, Caine V, Goez H The encounters and challenges of ethnography as a methodology in health research. Int J Qual. 2015;14:1–16.

[17] Cleland JA The qualitative orientation in medical education research. KJME. 2017;29:61–71. 28597869 10.3946/kjme.2017.53 PMC5465434

[18] Roper JM, Shapira J Ethnography in nursing research. Thousand Oaks(CA): Sage Publications; 2000.

[19] Blomberg J, Burrell M An ethnographic approach to design In: Jacko JA, editor. The human-computer interaction handbook: fundamentals, evolving technologies, and emerging applications. New York (NY): CRC; 2012 p. 1025–1052.

[20] Muecke MA On the evaluation of ethnographies In: Morse JM, editor. Critical issues in qualitative research methods. Thousand Oaks (CA): Sage; 1994 p. 187–209.

[21] Caine KJ, Davison CM, Stewart EJ Preliminary fieldwork: methodological reflections from northern Canadian research. Quali Res. 2009;9:489–513.

[22] Smith VJ Ethical and effective ethnographic research methods: a case study with Afghan refugees in California. J Empir Res Hum Res Ethics. 2009;4(3):59–72. 10.1525/jer.2009.4.3.59 19754236

[23] [cited 2018 9] Available from: http://www.cihr-irsc.gc.ca/e/29418.html

[24] Lavis JN, Robertson D, Woodside JM, et al How can research organizations more effectively transfer research knowledge to decision makers? Milbank Q. 2003;81:221–222. 12841049 10.1111/1468-0009.t01-1-00052 PMC2690219

[25] Clifford J, George EM Writing culture: the poetics and politics of ethnography. Berkeley (CL): University California Press; 1986.

[26] Wieringa S, Engebretsen E, Heggen K, et al Rethinking bias and truth in evidence-based health care. J Eval Clin Pract. 2018;24:930–938. 30079500 10.1111/jep.13010 PMC6175413

[27] Bryman A The research question in social research: what is its role? Int J Soc Res Methodol. 2007;10:5–20.

[28] Hulley SB, Cummings SR Designing clinical research. An epidemiologic approach. Baltimore: Williams & Williams; 1988.

[29] Hart E Getting started in oral traditions research. Occasional Papers of the Prince of Wales Northern Heritage Centre, No. 4 NT: Government of the Northwest Territories Yellowknife; 1995.

[30] Hammersley M, Atkinson P Ethnography: principles in Practice 2nd ed. London (UK): Routledge; 1983.

[31] Cruz EV, Higginbottom G The use of focused ethnography in nursing research. Nurse Res. 2013;20:36–43. 23520711 10.7748/nr2013.03.20.4.36.e305

[32] Creswell JW Research design: qualitative, quantitative, and mixed methods approaches (3rd ed.). Thousand Oaks (CA): Sage Publications; 2009.

[33] de Munck VC A micro-, meso-, and macro-level descriptive analysis of disputes within a social network. A study of household relations in a Sri Lankan community. Anthropos. 1994;89:85–94.

[34] Polit DF, Beck CT Nursing research: generating and assessing evidence for nursing practice. Philadelphia (PA): Lippincott Williams and Wilkins; 2012.

[35] Lincoln SY, Guba GE Naturalistic inquiry. California (USA): Sage Publications, Inc; 1985.

[36] Denzin NK Sociological methods: a sourcebook. New York (NY): McGraw-Hill; 1978.

[37] Geertz C Thick description: toward an interpretive theory of culture In: Geertz C, editor. Interpretation of cultures. Boston (USA): Basic Books; 1973 p. 3–30.

[38] Ponterotto JG Brief note on the origins, evolution and meaning of the qualitative research concept ‘thick description’. Qual Rep. 2006;11:538–554.

